# Malign Differentiation of a Large Buschke Loewenstein Tumor in Penis

**DOI:** 10.1055/s-0038-1635571

**Published:** 2018-03-28

**Authors:** Osman Akdag, Gokce Yildiran

**Affiliations:** 1 Department of Plastic, Reconstructive and Aesthetic Surgery, Selcuk University Medical Faculty, Konya, Turkey

**Keywords:** Buschke–Loewenstein tumor, condyloma acuminate, malignant transformation, penis

## Abstract

Buschke–Loewenstein tumor (BLT) is a giant condyloma acuminatum which is very rare and commonly affects the anogenital region. The malignant transformation and localization in penis is very rare. This article aims to present a case with BLT with malignant transformation in penis.

A 59-year-old male patient was referred, who has have been suffering from a cauliflower-like lesion in the penis for 8 years. Biopsies revealed a BLT with malignant transformation. The lesion was excised largely and reconstructed with local flaps and skin grafts.

Defined by Buschke and Loewenstein in 1925, giant condyloma acuminatum is a rare and important disorder because of the sexually transmitting capability.

The Buschke–Loewenstein tumor (BLT) is a sexually transmitted and very rare giant condyloma acuminatum. 1 2 This lesion is usually a large cauliflower-like tumor affecting the anogenital region. 2 3 Although it is a benign tumor that develops slowly, it has destructive and local aggressive features. 2 4 Also, it has a malignant differentiation potential which is mostly to the squamous cell carcinoma. 3 Several treatment options have been described in the literature for BLT, but how malignant transformation should be treated is controversial. 3 5 This article aims to present a very rare malignant transformation of a large penile BLT case and its treatment.

## Case

**Fig. 1:**
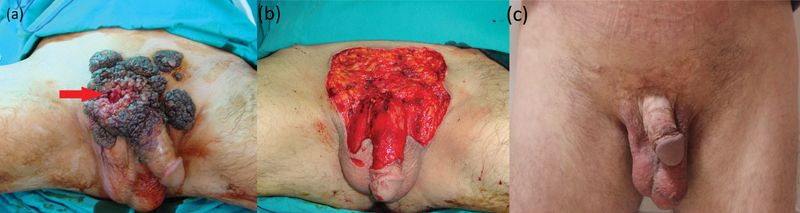
( **a** ) Preoperative view of 15 × 15 cm condyloma accuminata. Red arrow shows the malignant transformation area of this giant Buschke–Loewenstein tumor. ( **b** ) Intraoperative view of defect after the excision of tumor with 1 cm surgical margin. ( **c** ) Postoperative 6th year view of the patient.

A 59-year-old male patient was admitted to our clinic with a lesion starting from the penis and growing slowly for 8 years. The patient's human papillomavirus (HPV) and human immunodeficiency virus (HIV) tests were negative and had no known disease. Multiple biopsies were taken from the depressed and pink-red areas, which differed from the lesion in appearance (red arrow shows malignant transformation area), all of them were reported as the squamous cell carcinoma arising in the condyloma acuminatum of Buschke and Loewenstein ( Fig. 1a ). There was no inguinal pathological lymphadenopathy. The lesion was extensively excised with 1 cm intact surgical margin macroscopically ( Fig. 1b ). Areas other than the penile skin were repaired with split-thickness skin grafts, while penile skin defect was repaired with scrotal cutaneous flaps elevated from the scrotal skin to prevent the secondary contractions. Surgical borders were intact, and histopathologic reports showed a malignant transformation on the giant condyloma. The patient was followed for 6 years postoperatively and no recurrence was observed ( Fig. 1c ). Penile radix had no contracture and erectile function was normal.

## Discussion

BLT was described by Buschke and Loewenstein in 1925 as a giant condyloma acuminatum case of penile BLT. 3 6 Condyloma acuminatum is known to have a local destructive effect without metastasis. 7 It rarely progresses into squamous cell carcinoma. 1 2 Whether a partially ulcerated, bleeding mass is determined, squamous cell carcinoma differentiation of tumor may be suspected. Condyloma acuminata is associated with HPV infection and malignant transformation is common in HIV-positive patients. 7 The case differs with the absence of HPV and HIV infection and the reason of very rare malignant transformation.

In the literature, it is defined that BLT is commonly seen in the anogenital region. 8 9 Being settled in penis makes this case more special and rare.

The treatment options for condyloma acuminatum include cryosurgery, topical podophyllin, or trichloroacetic acid; however, these treatments are much more convenient for small lesions. 7 Because of being locally aggressive, destructive, and malignant transformation potency, exact therapy is the surgical excision. However, if there is a malignant transformation as in our case and to prevent the recurrences, excisions should be performed with large surgical margins. Penile BLT has a specialty because wide resections of penile skin are challenging to reconstruct. We did not prefer skin graft as the main reconstruction method to prevent penile contracture and maintain the erectile function. For this reason, we preferred local flaps elevated from the scrotum. Because of the benefit of excess scrotal skin, the donor site of the scrotal flaps could be repaired primarily, and donor site morbidity was reduced.

Since there is a possibility of malignant transformation, we recommend both radical surgical excision of condylomas in this area without fear and reconstruction with local flaps for penis and skin grafts for the suprapubic regions.

## Conclusion

Condyloma acuminata is rarely accompanied by malignancies. Also, reconstruction and management of this rare condition is crucial. A functional repair of a condyloma acuminata defect was presented. We think that this work will contribute to the literature.
